# Efficacy of the prophylactic use of octreotide for the prevention of complications after pancreatic resection: An updated systematic review and meta-analysis of randomized controlled trials

**DOI:** 10.1097/MD.0000000000007500

**Published:** 2017-07-21

**Authors:** Chunli Wang, Xin Zhao, Shengyi You

**Affiliations:** a Department of General Surgery, Tianjin Medical University General Hospital; b Nankai Clinical School, Tianjin Medical University, Tianjin, China.

**Keywords:** complications, meta-analysis, pancreatic resection, prophylactic octreotide

## Abstract

**Background:**

The use of octreotide prophylaxis in the prevention of complications after pancreatic resection remains controversial. The aim of this systematic review and meta-analysis was to evaluate the efficacy of octreotide prophylactic treatment to prevent complications after pancreatic resection.

**Methods:**

Five databases (PubMed, Medline, SinoMed, Embase, and Cochrane Library) were searched for eligible studies from 1980 to November 2016 with the limitation of human subjects and randomized controlled trials (RCTs). Data were extracted independently and were analyzed using RevMan statistical software version 5.3 (Cochrane Collaboration, http://tech.cochrane.org/revman/download). Weighted mean differences (WMDs), risk ratios (RRs), and 95% confidence intervals (CIs) were calculated. Cochrane Collaboration risk of bias tool was used to assess the risk of bias.

**Results:**

Twelve RCTs comprising 1902 patients were identified as eligible. The methodological quality of the trials ranged from low to moderate. A pooled analysis of effectiveness based on the data from each study revealed that octreotide could significantly reduce the rate of pancreatic fistula (PF) after pancreatic resection (RR = 0.75, 95% CI = 0.57–0.98, *P* = .04). The same findings were discovered in multicenter and European subgroups with a subgroup analysis; no obvious differences were noted in American, Asian, and single-center subgroup analyses. An equal effect was observed between the use or non-use of octreotide groups regarding mortality (RR = 1.24, 95% CI = 0.77–2.02, *P* = .38). Octreotide had no advantages in regards to mortality improvement. The total numbers of complications associated with the use or non-use of octreotide were similar (RR = 0.77, 95% CI = 0.58–1.03, *P* = .08). Among the high-risk group, octreotide was more effective in reducing complications (RR = 0.61, 95% CI = 0.46–0.82, *P* = .0009). Compared with the patients who did not receive prophylactic treatment, the patients who underwent pancreatic resection benefited from octreotide because it had better efficacy in preventing fluid collection and postoperative pancreatitis.

**Conclusion:**

The prophylactic use of octreotide is suitable for preventing postoperative complications, especially PF and fluid collection as well as postoperative pancreatitis. However, no obvious differences were noted regarding mortality. In view of the clinical heterogeneity and varying definitions of PF, whether these conclusions are broadly applicable should be further determined in future studies.

## Abstract 2

Supplemental Digital Content is available in the text

## 1 Introduction

In the pancreas, diseases that require surgical treatment primarily include pancreatic adenocarcinoma, benign tumor, and chronic pancreatitis. Early surgical treatment in chronic pancreatitis can not only improve the quality of life of patients to relieve pain and retain the internal and external secretion of pancreatic function but also effectively remove cancer risk factors.^[1]^ As surgical approaches to the treatment of pancreatic disease have undergone a transformation over the past few decades, they have become a relatively safe method for various benign and malignant pancreatic diseases, with mortality rates below 5%.^[2]^ However, patients undergoing surgical treatment also have a high incidence of complications; in particular, pancreatic fistula (PF) after pancreatic resection remains as high as 30% to 50%.^[3–5]^ PFs are the most serious and common complications after pancreatic surgery.^[6–8]^ Because these complications are mainly associated with exocrine pancreatic secretion, inhibiting the exocrine secretion of the pancreas is considered a suitable method to avoid PF development. Moreover, as early as 1979, Klempa et al^[9]^ noted that inhibition of pancreatic exocrine secretion could reduce the incidence of PF.

Octreotide (SMS 201–995), a long-acting octapeptide analog of somatostatin, was synthesized to have more specific, more potent, and longer-acting inhibitory effects than native somatostatin.^[10,11]^ In 1986, octreotide was considered useful in the prevention of postoperative complications after pancreatic resection.^[12]^ It can powerfully inhibit basal and stimulated exocrine pancreatic secretion, making it more advantageous for clinical applications.^[13]^ Octreotide has been recognized as one of the somatostatin analogs used in the prevention of PF after resection.^[14]^ However, some different results have also been obtained.^[15,16]^ Despite 30 years of octreotide clinical use in preventing postoperative complications, especially PF, evidence of the benefit of its use is still lacking, and there is currently no consensus regarding recommendations or guidelines. The efficacy of prophylactic octreotide in preventing postoperative complications remains speculative.

To further assess the existing evidence, we assessed the efficacy of prophylactic octreotide in the prevention of postoperative complications, particularly the formation of PF and mortality. We conducted an updated meta-analysis with a thorough search of the current literature to evaluate the efficacy of prophylactic octreotide for the prevention of postoperative complications after pancreatic resection.

## 2 Methods

Ethical approval or patient consent was not required since the present study was a review of previous published literature.

### 2.1 Search strategy and study selection criteria

A computerized search was conducted from 1980 to November 2016 with the PubMed, Medline, SinoMed, Embase, and Cochrane Library databases. The databases were queried for eligible literature using combinations of the following medical subject headings (MeSH): “pancreaticoduodenectomy or PD or pylorus-preserving pancreaticoduodenectomy or PPPD or pancreatic resection or pancreatectomy” and “octreotide or octreotide acetate or somatostatin analog” and “randomized controlled trial or controlled clinical trial or randomized or placebo or clinical trials as topic or randomly or trial.” The detailed search strategy for each database was provided (see supplemental content). The search was limited to human subjects. There was no language limitation. The titles and abstracts of potentially relevant studies identified by the computerized search were reviewed. Additionally, we reviewed abstracts from a conference of the Ihpba World Congress. Full-text articles were obtained for detailed evaluation, and eligible studies were included in the systematic review.

The inclusion criteria were the following: the study included outpatients who were of either sex, had a clinical and histological diagnosis of chronic pancreatitis, pancreatic adenocarcinoma, or other pancreatic-related benign tumor, and were undergoing elective pancreatic resections; octreotide should be administered as prophylaxis, with the aim of the trial being a comparison of the effectiveness of octreotide in preventing complications after pancreatic resection in the octreotide group and a placebo or no intervention in the control group; the method of administration should be subcutaneous, and outcomes included at least the incidence of postoperative PF, mortality, and other postoperative complications; and study designs should be randomized controlled trials (RCTs), including multicenter and single-center trials.

The exclusion criteria were the following: patient information data that were insufficiently clear; application of other drugs, such as different somatostatin analogs or therapies during the treatment.

### 2.2 Data collection and extraction

Two coauthors independently reviewed all titles and abstracts of the searched papers. Extracted data included the characteristics of the eligible studies, such as author, country, details of the study design, sample size, sex, mean age, interventions, incidences of postoperative PF, mortality, numbers of complications, and disease pathology. Discrepancies were resolved through discussion or with a third party to resolve conflicting evaluations. In 2 of the included studies, patients were stratified into high-risk (those with tumors in the pancreas or periampullary region) and low-risk (those with chronic pancreatitis) groups. Both the high-risk and low-risk groups had available data on PFs that were extracted and assessed in the study.

### 2.3 Outcome measures

The primary outcome was the incidence of PF after pancreatic surgery and mortality during the treatment. The analyses of overall occurrence of all grades PF (grades A, B, and C) and only to those having a clinical impact PF (only grades B and C) were conducted. Secondary outcomes were other postoperative complications, such as anastomosis leakage, abscess, fluid collection, shock, sepsis, pulmonary insufficiency, renal insufficiency, bleeding, and postoperative pancreatitis. Moreover, the adverse effects of the study drugs, had also been described.

### 2.4 Quality assessment and risk of bias

Two reviewers independently screened, extracted, and checked the research data to ensure consistency. The quality of trials that were designed with control and treatment groups was assessed using Review Manager (Version 5.3; The Cochrane Collaboration, Oxford, UK). The risk of bias for RCT studies was evaluated with Cochrane Collaboration Risk of Bias Tool. Seven parameters were used to evaluate the quality of each included study: random sequence generation, allocation concealment, blinding of participants and personnel, blinding of outcome assessment, incomplete outcome data, selective outcome reporting, and other risks. Items were judged as “low risk,” “unclear risk,” or “high risk.” Any disagreement was resolved by a discussion, and a consensus was reached.

### 2.5 Statistical methods

In the systematic review, meta-analysis was conducted using Review Manager 5.3 software (Cochrane Collaboration, http://tech.cochrane.org/revman/download). For dichotomous outcomes in the extracted data, risk ratios (RRs) and 95% confidence intervals (CIs) were calculated, and weighted mean differences (WMDs) and 95% CIs were used for continuous outcomes. Heterogeneity was assessed using the *Q* test and *I*^2^ test. Statistical significance was set at *P* < .05. If there was significant heterogeneity, *P* < .05 and *I*^2^ > 50%, if there was no significant heterogeneity, *P* ≥ .05 and *I*^2^ ≤ 50%. In view of the clinical and methodogical heterogeneity across the studies, if the same results were obtained under these 2 models, a random-effects model was a more appropriate choice. When the interquartile range (IQR) and median were given instead of the standard deviation (SD), we converted the data using Hozo algorithm to estimate the SD.^[17]^

### 2.6 Subgroup and sensitivity analyses

Subgroup analyses based on the different study designs (multicenter or single-center), the geographical location (Europe, America, or Asia), and the pathology of the disease (low-risk stratum or high-risk stratum) were performed with available data to access the efficacy of the octreotide prophylactic treatment to prevent complications after pancreatic resection. The high-risk stratum included patients suffering from tumors such as pancreatic cancer, periampullary cancer, and endocrine tumor, while the low-risk stratum included patients who had chronic pancreatitis. We also performed sensitivity analysis to assess the stability of the results and investigate the influence of each study by omitting a single study sequentially. Publication bias was showed by funnel plot.

## 3 Results

### 3.1 Data extraction

**Figure 1:**
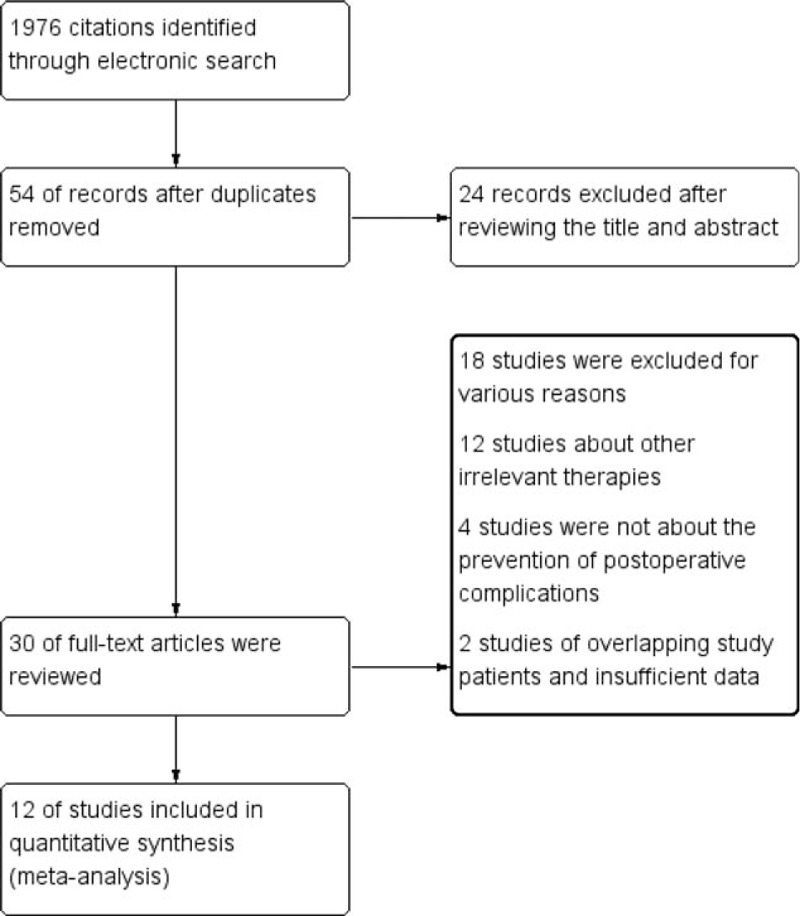
Flow diagram for the selection of randomized controlled trials included in the meta-analysis.

Of the 1976 citations identified based on a study of the subject and a summary of the literature, 1922 articles were excluded because of duplication. After reviewing the title and abstract of the remaining 54 studies, only 30 full-text studies were evaluated for further assessment, and 18 obviously irrelevant records were excluded. Finally, 12 clinical studies satisfied the inclusion requirements.^[18–29]^ A detailed study flow diagram is shown in Fig. 1.

### 3.2 Description of studies

All 12 of the assessed studies were in English. The meta-analysis involved a total of 1902 patients: 964 were randomized to the octreotide group, and 938 were randomized to the control group. Eight of the 12 studies were randomized placebo-controlled trials, and the remaining studies were RCTs of octreotide versus no treatment. Among the 12 identified studies, 6 were single-center trials conducted in the United States (1),^[18]^ Belgium (1),^[19]^ Switzerland (1),^[20]^ Spain (1),^[21]^ and India (2).^[22,23]^ The other 6 studies were multicenter trials conducted in Germany (3),^[24–26]^ Italy (1),^[27]^ the United States (1),^[28]^ and France (1).^[29]^ The mean age ranged from 47.0 to 69.0 years. The majority of patients enrolled in the 10 studies had standard clinical diagnoses of pancreatic adenocarcinoma, endocrine tumor, periampullary tumor, chronic pancreatitis, and other pancreatic diseases requiring surgical treatment.^[18–22,24,25,27–29]^ Only 2 studies enrolled patients who suffered only from chronic pancreatitis.^[23,26]^ The daily dose of octreotide ranged from 100 to 250 μg administered subcutaneously every 8 hours, and the duration of intervention ranged from 5 to 10 days. In addition, 2 included studies stratified patients into 2 groups, high-risk and low-risk patients, according to the characteristics of pancreatic pathology. Patients with tumors of the pancreas or periampullary region were in the high-risk group, and patients with chronic pancreatitis were in the low-risk group.^[24,25]^ All the included studies evaluated the incidence of PF, mortality, and other related complications, and we extracted relevant useful data to conduct our analyses. The characteristics of the included studies are presented in Table 1.

**Table 1:**
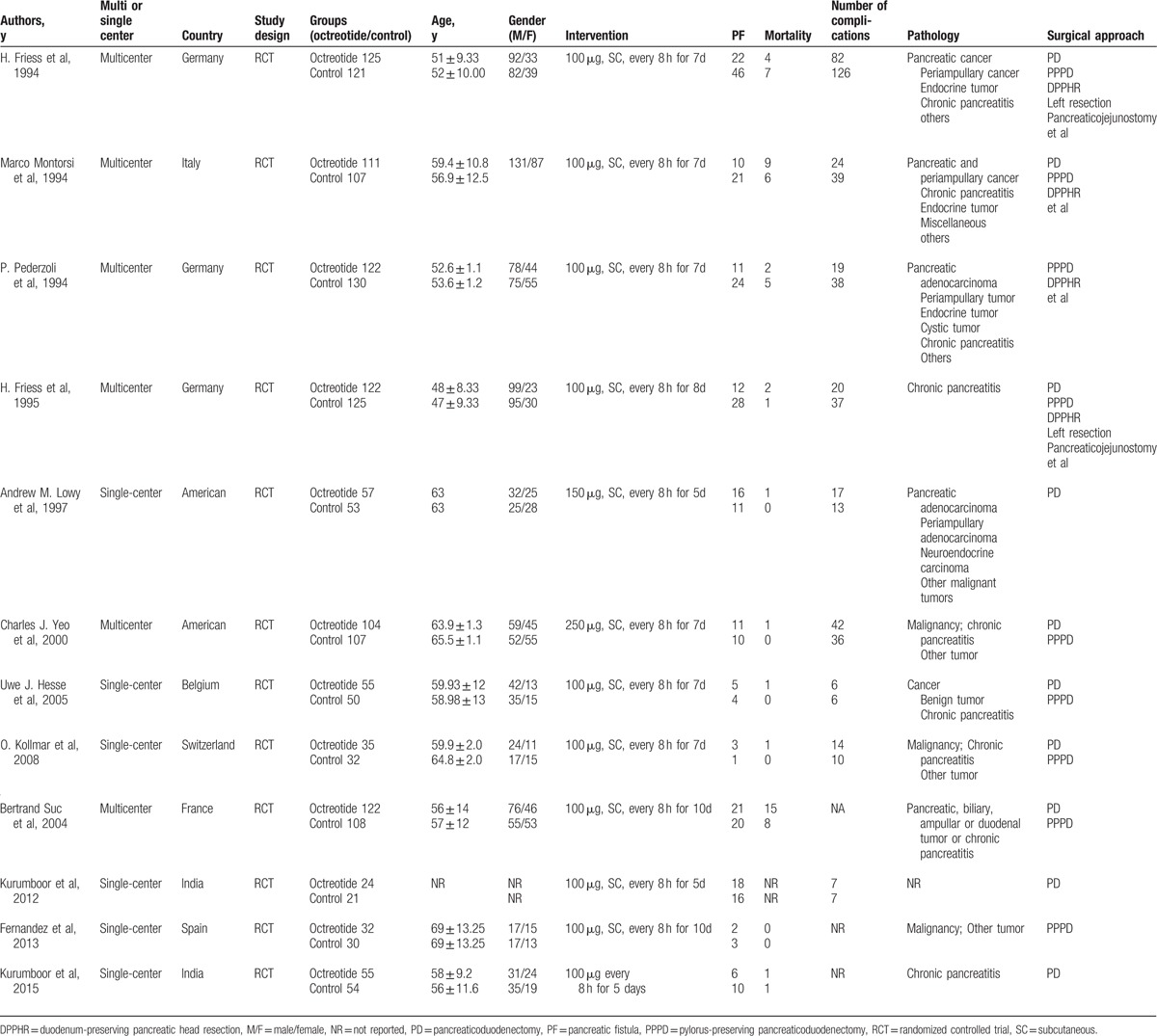
Characteristics of studies included and clinical outcomes of the study population.

### 3.3 Methodological assessment of study quality

**Figure 2:**
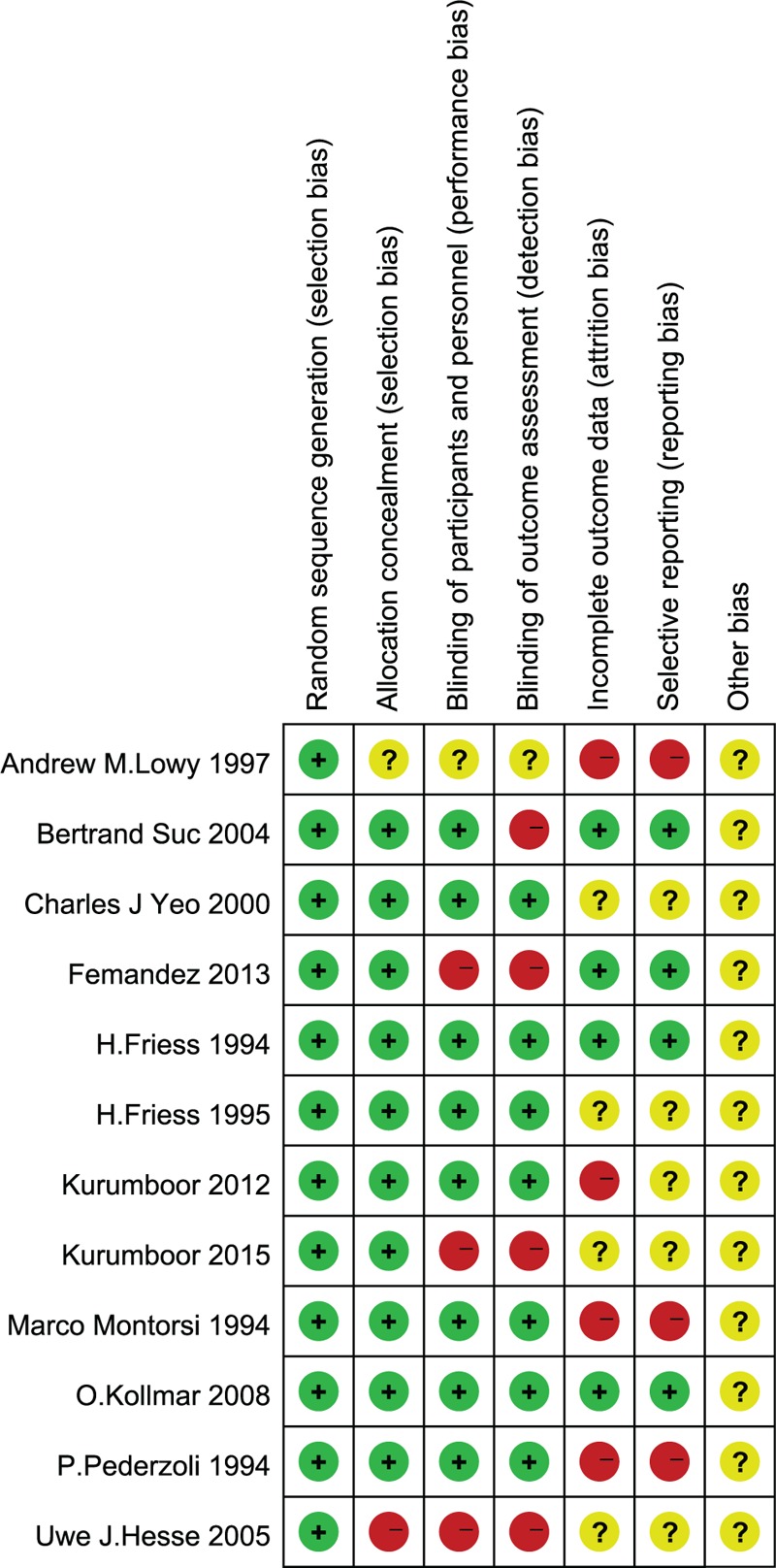
Risk of bias summary: this risk of bias tool incorporated the assessment of randomization (sequence generation and allocation concealment), blinding (participants and outcome assessors), incomplete outcome data, selective outcome reporting, and other risks of bias. The items were judged as “low risk,” “unclear risk,” or “high risk.” Red means “high risk,” green means “low risk,” and yellow means “unclear risk.”

**Figure 3:**
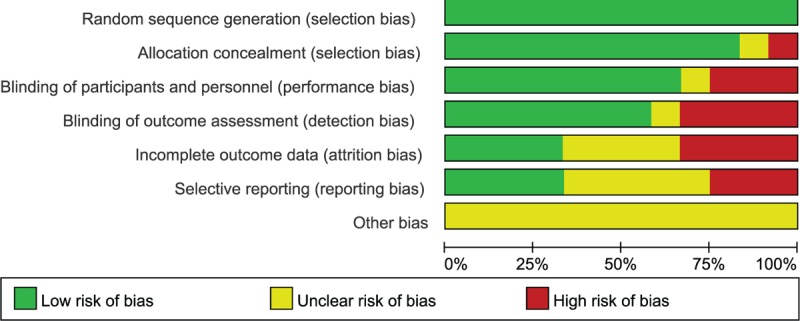
Risk of bias graph exhibiting the review of the authors’ judgments about each risk of bias item, presented as percentages across all included studies.

The Cochrane Collaboration tool was used to assess the risk of bias of the included studies. The methodological quality assessment of the 12 included studies is presented in Fig. 2. The quality of these studies was low to moderate. All identified studies were RCTs, and randomization was performed according to a computer-generated random list or by means of a randomly generated number pattern in a majority of the trials. Six of the included studies were double-blind placebo-controlled trials,^[20,24–28]^ 1 was a single-blind study,^[29]^ and 1 was an open-label trial^[19]^; the remaining 4 studies did not mention whether they used a method of blinding that may introduce measurement bias.^[18,21–23]^ The method of allocation concealment was not described in detail, giving rise to a high risk for selection and measurement bias. Thus, 6 out of 12 trials were single-center trials,^[18–23]^ and the other 6 studies were multicenter trials,^[24–29]^ which may also have been a source of bias. See Fig. 3.

### 3.4 Primary outcome: incidence of PF and mortality

**Figure 4:**
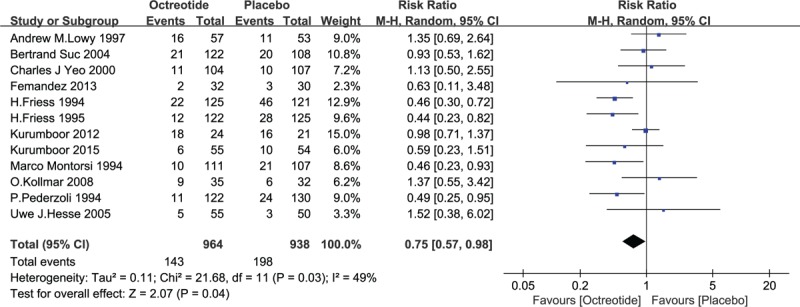
Forest plot of randomized controlled trials of prophylactic octreotide versus no intervention in pancreatic fistula. CI = confidence interval, RR = relative risk.

**Figure 5:**
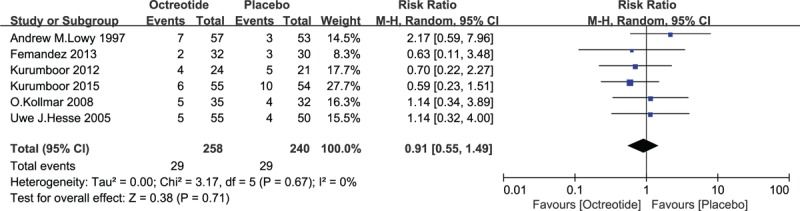
Forest plot of randomized controlled trials of prophylactic octreotide versus no intervention in clinically significant pancreatic fistula. CI = confidence interval, RR = relative risk.

From the aforementioned studies, a total of 341 patients suffered from PF (341/1902, 17.93%) after pancreatic resection: 143 PFs occurred in the octreotide group (143/964, 14.83%), and 198 occurred in the placebo group. Moderate heterogeneity among the studies was revealed (*I*^2^ = 49%), and the random-effects model was adopted in the analysis. The pooled RR was 0.75 (95% CI 0.57–0.98, *P* = .04). Grade B and C fistulas were identified as clinically significant PFs. Fifty-eight clinically significant PFs occurred (58/498, 11.65%): 29 in the octreotide group (29/258, 11.24%) and 29 in the placebo group (29/240, 12.08%). A pooled analysis revealed that there was no statistically significant difference between the 2 groups in the induction of clinically significant PF (RR = 0.91, 95% CI = 0.55–1.49, *P* = .71). See Figs. 4 and 5.

**Figure 6:**
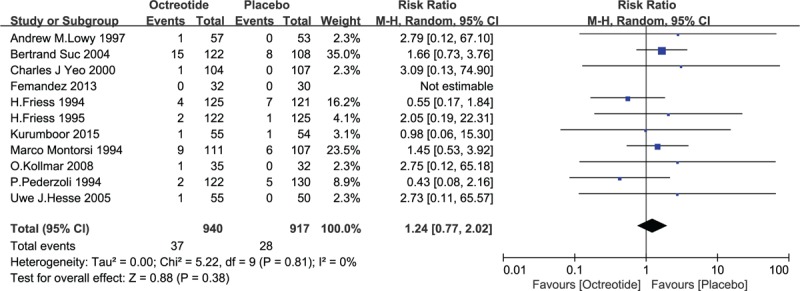
Forest plot of randomized controlled trials of prophylactic octreotide versus no intervention in mortality. CI = confidence interval, RR = relative risk.

All identified studies reported on mortality, except 1.^[22]^ Sixty-five deaths occurred (65/1857, 3.5%): 37 in the octreotide group (37/940, 3.9%), and 28 in the control group (28/917, 3.1%). A pooled analysis revealed that RR was 1.24 (95% CI 0.77–2.02, *P* = .38). No significant differences between the 2 groups were observed. See Fig. 6.

### 3.5 Secondary outcome: postoperative complications

**Figure 7:**
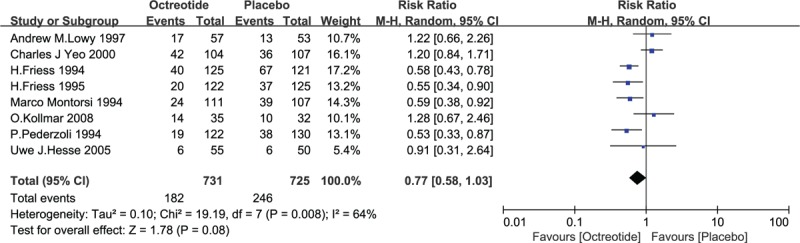
Forest plot of randomized controlled trials of octreotide versus no intervention in the incidence rate of complications. CI = confidence interval, RR = relative risk.

After pooling all the trials, 8 studies were found to contain relevant data on patients with complications, comprising a total of 1456 patients. Specifically, 182 out of 731 patients who were administered octreotide before the operation reported complications, and 246 out of 725 patients in the control group showed side effects. A heterogeneity test revealed significant heterogeneity among the studies (*I*^2^ = 64%); thus, the random-effects model was used. The pooled analysis under the random-effects model indicated that there was no significant difference in the incidence of complications between the octreotide and control groups (RR = 0.77, 95% CI = 0.58–1.03, *P* = .08). The finding that the upper confidence limit for the RR barely exceeded 1.0 and that the horizontal block lay to the left of the vertical line indicated that the administration of octreotide preoperatively may reduce the rate of complications. See Fig. 7.

A pooled analysis of the complications showed that there was no significant difference between the 2 groups in anastomosis leakage, abscess, shock, sepsis, pulmonary insufficiency, renal insufficiency, bleeding, wound infection, and delayed gastric emptying. However, the administration of octreotide preoperatively significantly reduced the rates of fluid collection. In the result of inducing postoperative pancreatitis, given that the upper confidence limit for the RR barely exceeds 1.0, and that the horizontal block lies to the left of the vertical line, it indicates that prophylactic treatment of octreotide could reduced the incidence of postoperative pancreatitis. The detailed complication results are shown in Table 2.

**Table 2:**
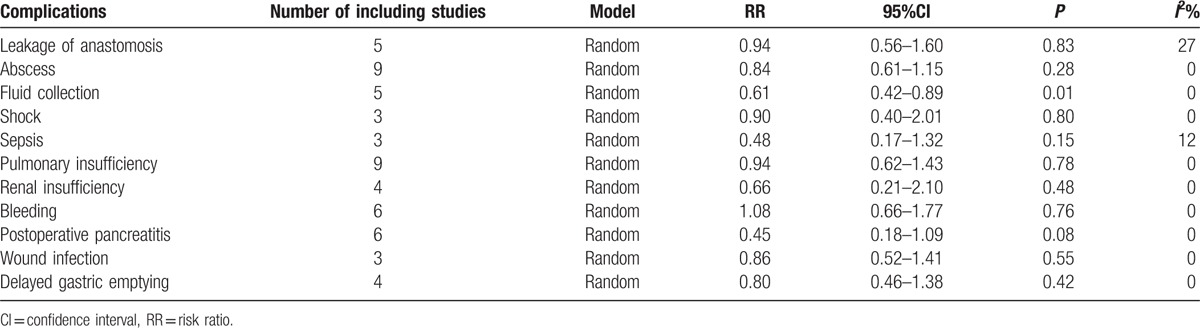
Results of complications of patients.

### 3.6 Adverse events

**Figure 8:**
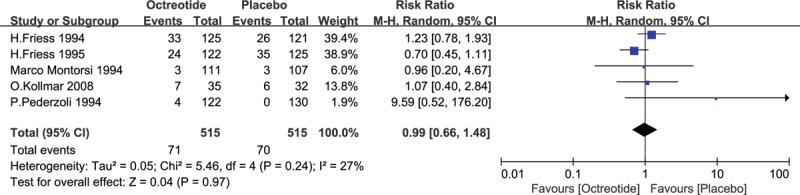
Forest plot of randomized controlled trials of the adverse effects to the study drugs (octreotide vs placebo). CI = confidence interval, RR = relative risk.

Five trials had records of incidence number of adverse events. The analysis under random-effect model pooled estimate of RR was 0.99 (95% CI: 0.66, 1.48), which showed no significant difference between 2 groups (*P* = .97). The relevant details were showed in Fig. 8.

### 3.7 Subgroup analysis

**Figure 9:**
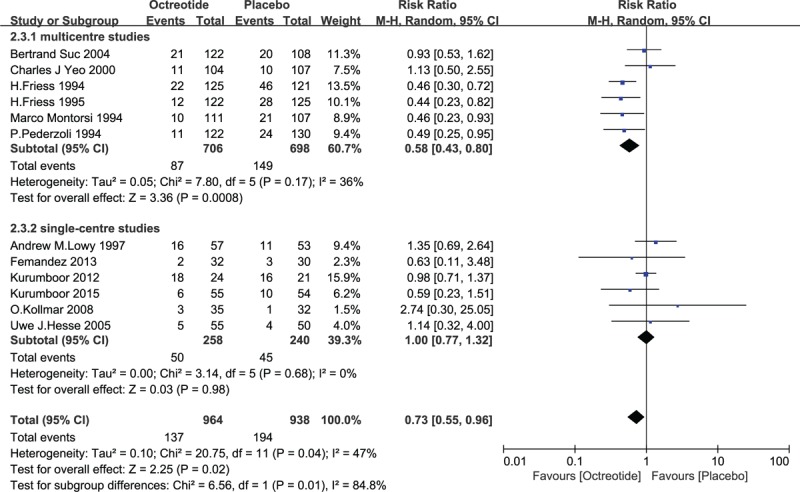
Forest plot of randomized controlled trials of octreotide versus no intervention with the type of study design (multicentre or singlecentre) in pancreatic fistula. CI = confidence interval, RR = relative risk.

Subgroup analysis was conducted according to the study design (multicenter or single-center). Six trials were multicenter trials, and the remaining 6 were single-center trials. In the multicenter studies, 87 out of 706 patients suffered PF with the octreotide prophylaxis, and 149 out of 698 patients suffered PF in the control group. There was a significant difference between the 2 groups in the induction of PF (RR = 0.58, 95% CI = 0.43–0.80, *P* = .0008). In the single-center subgroup, 50 out of 258 patients in the octreotide group had a PF postoperatively compared with 45 out of 240 in the control group. The pooled analysis under the random-effects model indicated that octreotide had no advantages in the prevention of postoperative PF (RR = 1.00, 95% CI = 0.77–1.32, *P* = .98). The results are discussed later. See Fig. 9.

**Figure 10:**
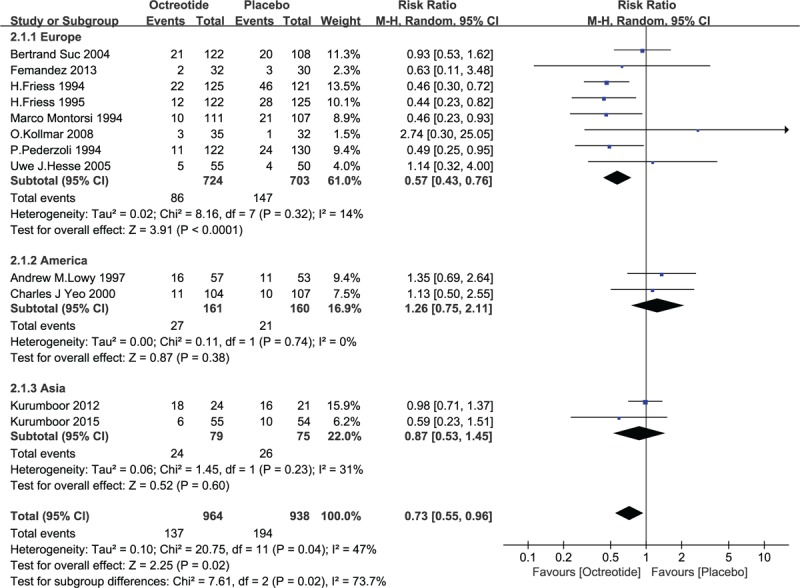
Forest plot of randomized controlled trials of octreotide versus no intervention with different continents (Europe or America or Asia) in pancreatic fistula. CI = confidence interval, RR = relative risk.

Studies from different continents (Europe, America, or Asia) were also analyzed as subgroups: 8 studies from Europe, 2 from North America, and 2 from Asia. In the European subgroup, the pooled analysis indicated that octreotide had advantages in the prevention of postoperative PF (RR = 0.57, 95% CI = 0.43–0.76, *P* < .0001). In the American and Asian subgroups, there were no statistically significant differences in the prevention of postoperative PF between the 2 groups (RR = 1.26, 95% CI = 0.75–2.11, *P* = .38; RR = 0.87, 95% CI = 0.53–1.45, *P* = .6). See Fig. 10.

**Figure 11:**
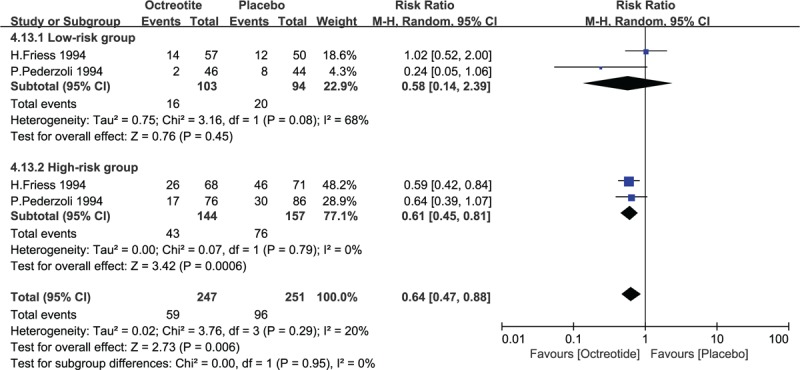
Forest plot of randomized controlled trials of octreotide vs. no intervention with the pathology of disease (low-risk and high-risk group) in the total number of patients with complications following pancreatic operation. CI = confidence interval, RR = relative risk.

The 2 included studies stratified patients into 2 groups: high-risk and low-risk groups.^[24,25]^ The subgroup meta-analysis of the low-risk and high-risk group patients had to be performed with available data regarding the total number of complications. The pooled analysis under random-effects in low-risk group with patient who suffered complications showed that there is no significant difference in the incidence of complications (RR = 0.58, 95% CI = 0.14–2.39, *P* = .45), while a significant difference in the incidence of complications in patients in high-risk group (RR = 0.61, 95% CI = 0.45–0.81, *P* = .0006). See Fig. 11.

### 3.8 Sensitivity analysis and publication bias

We performed a sensitivity analysis for assessing stability of pooled results. Among the most studies, the observed significant results were not obviously altered after sequentially omitting each study. In the pooled results comparing incidence of PF, after excluding the Fiess H study,^[24]^ the heterogeneity decreased significantly (RR = 0.80, 95% CI = 0.61–1.05, *P* = .11, *I*^2^ = 38%), and showed that there is no significant different in preventing the incidence of PF between 2 groups. So it was regarded as a result of heterogeneity. Similarly, other 4 studies^[23,25–27]^ were considered as the source of heterogeneity because the heterogeneity significantly changed and showed that there is no significant different in preventing the incidence of PF between 2 groups by excluding each of these studies in the pooled results comparing incidence of PF. Moreover, of the 12 studies evaluated, 6 studies used a double-blind method,^[20,24–28]^ 1 adopted a single-blind method,^[29]^ and 4 did not mention the blinding method.^[18,21–23]^ One was a single-blind study^[29]^ and 1 was an open-label trial.^[19]^ Therefore, a sensitivity analysis was conducted to determine whether the exclusion of this study would change the result. Exclusion of this study from the meta-analysis did not substantially influence the results.

**Figure 12:**
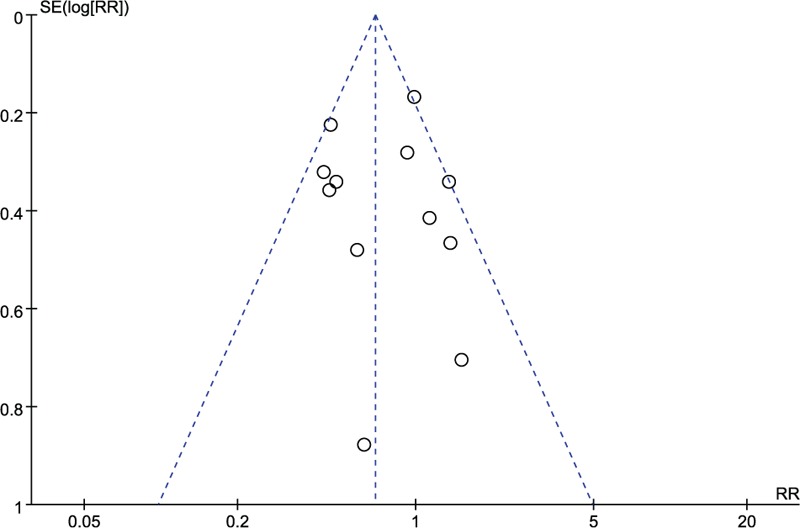
Funnel plots of randomized controlled trials of octreotide versus no intervention for outcome of pancreatic fistula. RR = risk ratio, SE = standard error.

A funnel plot of randomized controlled trials reporting PF outcomes is shown in Fig. 12. Publication bias may exists, but was not apparent. The result was discussed later.

## 4 Discussion

### 4.1 Summary of the main results

PF remains the most frequent complication after pancreatic resection.^[30]^ Some trials in the literature revealed that octreotide prophylaxis could significantly reduce the rate of PF.^[24–27,31]^ However, several groups of investigators evaluated the octreotide prophylactic and reported no statistical benefit for patients who underwent pancreatic resection.^[18,28,29,32,33]^ However, the results were quite conflicting.

This was an updated systematic review and meta-analysis of RCTs to assess the efficacy of octreotide prophylactic use for the prevention of complications after pancreatic resection. Octreotide could significantly reduce the rate of PF after resection. Additionally, the same findings were discovered in multicenter trials and the European subgroup by conducting subgroup analysis. Considering that 5 out of 6 trials, including multicenter RCTs, were from Europe, there is no doubt that similar results may be obtained. However, contradictory results were found in the remaining 6 single-center studies. These differences may be due to the experience level of the surgeon, the type of anastomosis, or the quality of the tissue. With the technical surgical improvements, the incidence of PF after PD has been successfully reduced.^[34,35]^ The type of surgery could influence the rate of PF development.

The grading of PF with grades A, B, and C has gained widespread acceptance which were defined according to the clinical impact on patients hospital course.^[36]^ Grade A postoperative pancreatic fistula was called a “biochemical leak,” because it has no clinical importance. The analyses of overall occurrence of all grades PF (grades A, B, and C) and only to those having a clinical impact PF (only grades B and C) were conducted. In our study, 6 trials compared the use of octreotide and reported clinically significant PFs using the International Study Group of Pancreatic Fistula (ISGPF) definition while demonstrated no difference in the incidence of clinically significant PF with or without the use of drugs.^[18–23]^ As for the result of clinically significant PF (grades B and C), there is no significant difference between octreotide and placebo groups. Considering that clinically significant PF may be closely related to the patient's surgical procedure, surgeon's technique, and the disease itself. Grade A postoperative pancreatic fistula is redefined and called a “biochemical leak,” because it has no clinical importance and is no longer referred to a true pancreatic fistula in the 2016 update of the International Study Group (ISGPS) definition and grading of postoperative pancreatic fistula.^[37]^ Therefore, we concluded that the occurrence of grade A PF is closely related to whether prophylactic use of octreotide is involved. In summary, our results support octreotide's benefit in avoiding the incidence of PF.

The definition of a PF varied in the 12 included studies and should be discussed. In this meta-analysis, we defined a PF as any volume with an amylase-rich fluid content of more than 3 times the serum level, exceeding 10 mL per 24 hours for more than 3 days. Fernandez et al^[20]^ and Kurumboor et al^[21]^ adopted the same definition, as did a trial in 1995.^[27]^ However, Yeo et al^[28]^ adopted a more conservative definition (>50 mL per 24 hours for more than 10 days or radiological pancreatic anastomosis disruption); this variation in definition may affect the results.

No significant difference in the rate of mortality was observed between the 2 groups. Although the 2 high-risk groups reached similar results, the *P* value was close to .05, and the horizontal block was located to the left of the vertical line. This result indicates a trend toward a decrease in mortality among patients suffering from pancreatic tumors. A study with a larger sample size would demonstrate the clinical implication of this difference.

An evaluation of the number of complications after pancreatic resection between the 2 groups revealed that there was no significant difference in the complication rate between the presence and absence of octreotide treatment. Studies by Friess et al^[26]^ and Kurumboor et al^[23]^ recruited only patients who suffered from chronic pancreatitis and indicated that octreotide had significant advantages in reducing the rate of complications. Thus, the pathology of pancreatic disease and the characteristics of the pancreatic parenchyma influence the incidence rate of complications.

As for the result of the adverse effects to the study drugs, there were no significant different between octreotide and placebo in induce adverse effects. A study reported that 59 patients (24 with octreotide, 35 with placebo) were observed which have side-effects during the study medication. Among these patients 43 patients (18 with octreotide, 25 with placebo) suffered some pain, burning or erythema at the injection site, and these effects did not require discontinuation of the treatment.^[26]^ Other events such as nausea, vomiting, heartburn, diarrhoea, intestinal cramps, dysopia, and disturbance of coagulation. And these effects did not require discontinuation of the treatment as well. In Kollmar O study,^[20]^ showed that 7 and 6 patients experienced delayed gastric emptying (DGE) with octreotide and placebo, respectively. This finding was not statistically significant. So, we speculated that DGE is one of surgical complications and may be not associated with the use of octreotide. The direct influence of surgical complications on DGE has been described in the previous studies.^[38,39]^ In Montorsi M study,^[27]^ 6 out of 218 patients experienced symptoms (nausea, vomiting, and diarrhea) possibly related to pharmacologic treatment (3 with octreotide, 3 with placebo). As well as the adverse events reported in other 3 including studies,^[24,25,18]^ none of these symptoms necessitate discontinuation of the treatment.

### 4.2 Comparison with previous studies

Given the widespread application of octreotide, RCTs assessed its efficacy in preventing complications after pancreatic resection directly. A study by Closset et al^[40]^ comparing somatostatin and octreotide proved that both somatostatin and octreotide have comparable efficacy in the prevention of complications after pancreatectomy. The function of octreotide in reducing fistula formation and promoting fistula closure is associated with 2 primary mechanisms: the inhibition of exocrine pancreatic secretion and the hardening of pancreatic tissue to facilitate safer anastomosis.^[41]^ A meta-analysis performed by Alghamdi et al^[42]^ summarized 7 RCTs and revealed that octreotide is associated with a significant reduction in the incidence of PF after pancreatic surgery, and no significant difference in postoperative mortality was observed. The results of the subgroup analysis according to the type of study design were consistent with our findings. A similar conclusion was also obtained by Li-Ling and Irving^[43]^ and Gurusamy et al,^[44]^ indicating that octreotide administration could reduce postoperative complications, particularly PFs, but could not reduce mortality. Different results have been summarized as well, showing that there is no decrease in the rate of PFs following octreotide administration after pancreatic resection.^[15,16]^ One recently completed comprehensive review of the use of somatostatin analogs in the prevention of postoperative complications identified 15 RCTs involving 1352 patients and demonstrated that octreotide had no influence on the incidence of PF.^[45]^ This study provided a relatively comprehensive evidence that prophylactic treatment with somatostatin or pasireotide have a potential role in reducing PF, while octreotide had no influence on the incidence of PF. As for the discrepancy between their and our findings, the potential clinical and methodological heterogeneity should be considered. The different search strategy and inclusion criteria may be attributed to the discrepancy. In Jin et al^[45]^ study, a subgroup analysis of patients divided into low-risk and high-risk group (according to the different nature of pancreatic disease) cannot be performed because of limited data. However, in our study the subgroup analysis of patients in low-risk and high-risk group were available. In addition, 2 other subgroup analysis were conducted according to study design and geographical, which provided more comprehensive evidence about prophylactic use of octreotide have benefit to avoid PF. This is one advantages of our study. Comparing with Jin et al study which evaluated prophylactic somatostatin analogues (somatostatin, pasireotide, and octreotide) in PD, our study investigated the effect of prophylactic octreotide on postoperative complications such as PF, mortality, anastomosis leakage, abscess, fluid collection, shock, sepsis, pulmonary insufficiency and so on which may provide more comprehensive and targeted information in evaluating the study drugs. According to the guidance of Cochrane Handbook, unpublished articles were involved in this meta-analysis what the previous article lacks may introduce publication bias. Rosenberg et al^[46]^ suggested that compared with a placebo, octreotide is a dominant treatment strategy. The prophylactic use of octreotide is a cost-effective strategy for patients undergoing pancreatic resection, especially those patients who are at high-risk for developing complications. Because only double-blind, randomized, controlled clinical trials were recruited in this meta-analysis, the results were more reliable. Another cost-effectiveness comparison of octreotide and pasireotide prophylactics for the prevention of fistula after pancreatic surgery yielded a similar conclusion.^[47]^

To the best of our knowledge, this is an updated systematic review and meta-analysis designed to evaluate the prophylactic treatment of octreotide to prevent complications after pancreatic resection. To provide more evidence for clinical decision-making, this study incorporated updated RCTs with a more detailed subgroup analysis (i.e., different study designs, geographical locations, and disease pathologies) that assessed the main results of postoperative PF in addition to mortality and the total number of complications (i.e., anastomosis leakage, abscess, fluid collection, shock, sepsis, pulmonary insufficiency, renal insufficiency, bleeding, and postoperative pancreatitis) with the available data to assess the efficacy of octreotide in preventing complications after pancreatic resection. The lack of these assessments was a limitation of our previous report. Moreover, some different comprehensive results were also observed; these findings were compared with the latest meta-analysis. Our study included more RCTs and performed subgroup analyses based on the study design, geographical location, and disease pathology. And a funnel plot was made to reveal the publication bias.

### 4.3 Limitations of the study

Despite a comprehensive analysis, certain limitations of this meta-analysis should be discussed. First, the most important limitation is the scarcity of high-quality, multicenter, large-sample standard RCTs that directly assess the efficacy of octreotide. Second, the PFs in each study were assessed by different definitions, potentially inducing inevitable bias. Third, the occurrence of postoperative complications was related to many factors, such as operative technique, surgeon experience, tissue quality, hospital volume, total perenteral nutrition, and other medical therapies, making the database rather imprecise. Although funnel plot is still a widely used method to detect publication bias, it's limitations should be aware. For example, change of metrics would change the shape of the plot; true heterogeneity and poor methodological quality could also lead to an asymmetric plot.^[48,49]^ Furthermore, Hozo algorithm was adopted for the parts of the included literature that did not directly provide means and SDs, which may have introduced bias. Moreover, different surgical procedures of pancreatic disease also affect the incidence of complications.^[50]^ Clinical and methodological heterogeneity was seen in several parameters in the meta-analysis, given the variation in surgical techniques, patient composition, and preferences among different centers. Unfortunately, limited data are available on the cost and financial implications of octreotide use. In view of the heterogeneity, more large, high-quality clinical trials that evaluate the efficacy of octreotide should be conducted, and more detailed analyses, such as analyses of financial constraints and safety tolerance, should be performed to strengthen the reliability of these conclusions.

## 5 Conclusion

The prophylactic use of octreotide is recommended, particularly for the prevention of postoperative complications associated with pancreatic fistula and fluid collection as well as postoperative pancreatitis in patients undergoing pancreatic resection. However, no obvious differences were noted regarding mortality. Further studies are warranted to confirm the results of this meta-analysis and to define which patient subgroups may benefit the most from prophylactic octreotide administration.

## Supplementary Material

Supplemental Digital Content

## References

[1] Ahmed AU Issa Y Bruno MJ Early surgery versus optimal current step-up practice for chronic pancreatitis (ESCAPE): design and rationale of a randomized trial. BMC Gastroenterol 2013;13:49. 23506415 10.1186/1471-230X-13-49 PMC3610165

[2] Winter JM Cameron JL Campbell KA 1423 pancreaticoduodenectomies for pancreatic cancer: a single-institution experience. J Gastrointest Surg 2006;10:1199–210. 1210–1211. 17114007 10.1016/j.gassur.2006.08.018

[3] Kamisawa T Wood LD Itoi T Pancreatic cancer. Lancet 2016;388:73–85. 26830752 10.1016/S0140-6736(16)00141-0

[4] Yeo CJ Cameron JL Sohn TA Six hundred fifty consecutive pancreaticoduodenectomies in the 1990s: pathology, complications, and outcomes. Ann Surg 1997;226:248–57. 257–260. 9339931 10.1097/00000658-199709000-00004 PMC1191017

[5] Bartoli FG Arnone GB Ravera G Pancreatic fistula and relative mortality in malignant disease after pancreaticoduodenectomy. Review and statistical meta-analysis regarding 15 years of literature. Anticancer Res 1991;11:1831–48. 1685076

[6] Warshaw AL Swanson RS Pancreatic cancer in 1988. Possibilities and probabilities. Ann Surg 1988;208:541–53. 2461172 10.1097/00000658-198811000-00001 PMC1493791

[7] McGuire GE Pitt HA Lillemoe KD Reoperative surgery for periampullary adenocarcinoma. Arch Surg 1991;126:1205–10. 1210–1212. 1681794 10.1001/archsurg.1991.01410340043007

[8] Schirmer WJ Rossi RL Braasch JW Common difficulties and complications in pancreatic surgery. Surg Clin North Am 1991;71:1391–417. 1948580 10.1016/s0039-6109(16)45596-9

[9] Klempa I Schwedes U Usadel KH [Prevention of postoperative pancreatic complications following duodenopancreatectomy using somatostatin]. Chirurg 1979;50:427–31. 477469

[10] Bauer W Briner U Doepfner W SMS 201-995: a very potent and selective octapeptide analogue of somatostatin with prolonged action. Life Sci 1982;31:1133–40. 6128648 10.1016/0024-3205(82)90087-x

[11] Pless J Bauer W Briner U Chemistry and pharmacology of SMS 201-995, a long-acting octapeptide analogue of somatostatin. Scand J Gastroenterol Suppl 1986;119:54–64. 2876507 10.3109/00365528609087432

[12] Kohler E Beglinger C Dettwiler S Effect of a new somatostatin analogue on pancreatic function in healthy volunteers. Pancreas 1986;1:154–9. 2437563 10.1097/00006676-198603000-00008

[13] Kemmer TP Malfertheiner P Buchler M Inhibition of human exocrine pancreatic secretion by the long-acting somatostatin analogue octreotide (SMS 201-995). Aliment Pharmacol Ther 1992;6:41–50. 1371938 10.1111/j.1365-2036.1992.tb00543.x

[14] Hesse U Ysebaert D de Hemptinne B Role of somatostatin-14 and its analogues in the management of gastrointestinal fistulae: clinical data. Gut 2001;49 (Suppl):v11–21. 10.1136/gut.49.suppl_4.iv11 PMC1766896 11878789

[15] Drymousis P Pai M Spalding D Is octreotide beneficial in patients undergoing pancreaticoduodenectomy? Best evidence topic (BET). Int J Surg 2013;11:779–82. 23800512 10.1016/j.ijsu.2013.06.013

[16] Gans SL van Westreenen HL Kiewiet JJ Systematic review and meta-analysis of somatostatin analogues for the treatment of pancreatic fistula. Br J Surg 2012;99:754–60. 22430616 10.1002/bjs.8709

[17] Hozo SP Djulbegovic B Hozo I Estimating the mean and variance from the median, range, and the size of a sample. BMC Med Res Methodol 2005;5:13. 15840177 10.1186/1471-2288-5-13 PMC1097734

[18] Lowy AM Lee JE Pisters PW Prospective, randomized trial of octreotide to prevent pancreatic fistula after pancreaticoduodenectomy for malignant disease. Ann Surg 1997;226:632–41. 9389397 10.1097/00000658-199711000-00008 PMC1191125

[19] Hesse UJ DeDecker C Houtmeyers P Prospectively randomized trial using perioperative low-dose octreotide to prevent organ-related and general complications after pancreatic surgery and pancreatico-jejunostomy. World J Surg 2005;29:1325–8. 16132406 10.1007/s00268-005-7546-1

[20] Kollmar O Moussavian MR Richter S Prophylactic octreotide and delayed gastric emptying after pancreaticoduodenectomy: results of a prospective randomized double-blinded placebo-controlled trial. Eur J Surg Oncol 2008;34:868–75. 18299182 10.1016/j.ejso.2008.01.014

[21] Fernandez-Cruz L Jimenez CE Taura P Prospective randomized trial of the effect of octreotide on pancreatic juice output after pancreaticoduodenectomy in relation to histological diagnosis, duct size and leakage. HPB (Oxford) 2013;15:392–9. 23557411 10.1111/j.1477-2574.2012.00608.x PMC3633042

[22] K Prakash, NP Kamalesh, K Pramil, et al. A prospective randomized controlled trial on use of octreotide in patients with soft pancreas undergoing pancreaticoduodenectomy: interim analysis. Ihpba World Congress; 2012.

[23] Kurumboor P Palaniswami KN Pramil K Octreotide does not prevent pancreatic fistula following pancreatoduodenectomy in patients with soft pancreas and non-dilated duct: a prospective randomized controlled trial. J Gastrointest Surg 2015;19:2038–44. 26302879 10.1007/s11605-015-2925-x

[24] Fiess H Klempa I Hermanek P Prophylaxis of complications after pancreatic surgery: results of a multicenter trial in Germany. Digestion 1994;55 (Suppl):35–40. 8132135 10.1159/000201187

[25] Pederzoli P Bassi C Falconi M Efficacy of octreotide in the prevention of complications of elective pancreatic surgery. Italian Study Group. Br J Surg 1994;81:265–9. 8156354 10.1002/bjs.1800810237

[26] Friess H Beger HG Sulkowski U Randomized controlled multicentre study of the prevention of complications by octreotide in patients undergoing surgery for chronic pancreatitis. Br J Surg 1995;82:1270–3. 7552016 10.1002/bjs.1800820938

[27] Montorsi M Zago M Mosca F Efficacy of octreotide in the prevention of pancreatic fistula after elective pancreatic resections: a prospective, controlled, randomized clinical trial. Surgery 1995;117:26–31. 7809832 10.1016/s0039-6060(05)80225-9

[28] Yeo CJ Cameron JL Lillemoe KD Does prophylactic octreotide decrease the rates of pancreatic fistula and other complications after pancreaticoduodenectomy? Results of a prospective randomized placebo-controlled trial. Ann Surg 2000;232:419–29. 10973392 10.1097/00000658-200009000-00014 PMC1421155

[29] Suc B Msika S Piccinini M Octreotide in the prevention of intra-abdominal complications following elective pancreatic resection: a prospective, multicenter randomized controlled trial. Arch Surg 2004;139:288–94. 295. 15006886 10.1001/archsurg.139.3.288

[30] Schlitt HJ Schmidt U Simunec D Morbidity and mortality associated with pancreatogastrostomy and pancreatojejunostomy following partial pancreatoduodenectomy. Br J Surg 2002;89:1245–51. 12296891 10.1046/j.1365-2168.2002.02202.x

[31] Gouillat C Gigot JF Pancreatic surgical complications—the case for prophylaxis. Gut 2001;49 (Suppl):v32–9. 10.1136/gut.49.suppl_4.iv29 PMC1766893 11878792

[32] Hesse UJ De Decker C Houtmeyers P Prospectively randomized trial using perioperative low dose octreotide to prevent organ related and general complications following pancreatic surgery and pancreatico-jejunostomy. Acta Chir Belg 2005;105:383–7. 16184721 10.1080/00015458.2005.11679741

[33] Barnett SP Hodul PJ Creech S Octreotide does not prevent postoperative pancreatic fistula or mortality following Pancreaticoduodenectomy. Am Surg 2004;70:222–6. 227. 15055845

[34] Motoi F Egawa S Rikiyama T Randomized clinical trial of external stent drainage of the pancreatic duct to reduce postoperative pancreatic fistula after pancreaticojejunostomy. Br J Surg 2012;99:524–31. 22497024 10.1002/bjs.8654

[35] Topal B Fieuws S Aerts R Pancreaticojejunostomy versus pancreaticogastrostomy reconstruction after pancreaticoduodenectomy for pancreatic or periampullary tumours: a multicentre randomised trial. Lancet Oncol 2013;14:655–62. 23643139 10.1016/S1470-2045(13)70126-8

[36] Bassi C Dervenis C Butturini G Postoperative pancreatic fistula: an international study group (ISGPF) definition. Surgery 2005;138:8–13. 16003309 10.1016/j.surg.2005.05.001

[37] Bassi C Marchegiani G Dervenis C The 2016 update of the International Study Group (ISGPS) definition and grading of postoperative pancreatic fistula: 11 years after. Surgery 2017;161:584–91. 28040257 10.1016/j.surg.2016.11.014

[38] Kimura F Suwa T Sugiura T Sepsis delays gastric emptying following pylorus-preserving pancreaticoduodenectomy. Hepatogastroenterology 2002;49:585–8. 11995503

[39] van Berge HM van Gulik TM DeWit LT Delayed gastric emptying after standard pancreaticoduodenectomy versus pylorus-preserving pancreaticoduodenectomy: an analysis of 200 consecutive patients. J Am Coll Surg 1997;185:373–9. 9328386 10.1016/s1072-7515(97)00078-1

[40] Closset J Journe S Mboti F Randomized controlled trial comparing somatostatin with octreotide in the prevention of complications after pancreatectomy. Hepatogastroenterology 2008;55:1818–23. 19102400

[41] Belyaev O Polle C Herzog T Effects of intra-arterial octreotide on pancreatic texture: a randomized controlled trial. Scand J Surg 2013;102:164–70. 23963030 10.1177/1457496913490457

[42] Alghamdi AA Jawas AM Hart RS Use of octreotide for the prevention of pancreatic fistula after elective pancreatic surgery: a systematic review and meta-analysis. Can J Surg 2007;50:459–66. 18053374 PMC2386209

[43] Li-Ling J Irving M Somatostatin and octreotide in the prevention of postoperative pancreatic complications and the treatment of enterocutaneous pancreatic fistulas: a systematic review of randomized controlled trials. Br J Surg 2001;88:190–9. 11167865 10.1046/j.1365-2168.2001.01659.x

[44] Gurusamy KS Koti R Fusai G Somatostatin analogues for pancreatic surgery. Cochrane Database Syst Rev 2012;CD008370. 22696379 10.1002/14651858.CD008370.pub2

[45] Jin K Zhou H Zhang J Systematic review and meta-analysis of somatostatin analogues in the prevention of postoperative complication after pancreaticoduodenectomy. Dig Surg 2015;32:196–207. 25872003 10.1159/000381032

[46] Rosenberg L MacNeil P Turcotte L Economic evaluation of the use of octreotide for prevention of complications following pancreatic resection. J Gastrointest Surg 1999;3:225–32. 10481115 10.1016/s1091-255x(99)80064-x

[47] Welsch T Mussle B Distler M Cost-effectiveness comparison of prophylactic octreotide and pasireotide for prevention of fistula after pancreatic surgery. Langenbecks Arch Surg 2016;401:1027–35. 27233242 10.1007/s00423-016-1456-6

[48] Lau J Ioannidis JP Terrin N The case of the misleading funnel plot. BMJ 2006;333:597–600. 16974018 10.1136/bmj.333.7568.597 PMC1570006

[49] Sterne JA Sutton AJ Ioannidis JP Recommendations for examining and interpreting funnel plot asymmetry in meta-analyses of randomised controlled trials. BMJ 2011;343:d4002. 21784880 10.1136/bmj.d4002

[50] Zhao X Cui N Wang X Surgical strategies in the treatment of chronic pancreatitis: an updated systematic review and meta-analysis of randomized controlled trials. Medicine (Baltimore) 2017;96:e6220. 28248878 10.1097/MD.0000000000006220 PMC5340451

