# Supplementary material for: Efficacy of the prophylactic use of octreotide for the prevention of complications after pancreatic resection: An updated systematic review and meta-analysis of randomized controlled trials
Source: Medicine (Baltimore). 2017 Jul 21;96(29):e7500. doi: 10.1097/MD.0000000000007500 (PMC5521901; doi:10.1097/MD.0000000000007500)
Supplement: Supplemental Digital Content [file medi-96-e7500-s001.doc]

The contents of appendix were as follows:

Pubmed search from 1980 to November 2016 under the search words: (((pancreaticoduodenectomy* OR PD* OR pylorus-preserving pancreaticoduodenectomy* OR PPPD*) AND (pancreatic resection*)) OR pancreatectomy[MeSH]) AND (“octreotide”[MeSH] OR “octreotide acetate” OR somatostatin analog) AND (randomized controlled trial [pt] OR controlled clinical trial [pt] OR randomized [tiab] OR placebo [tiab] OR clinical trials as topic [mesh: noexp] OR randomly [tiab] OR trial [ti])

MEDLINA search from 1980 to November 2016 under the search words: (((pancreaticoduodenectomy* OR PD* OR pylorus-preserving pancreaticoduodenectomy* OR PPPD*) AND (pancreatic resection*)) OR pancreatectomy[MeSH]) AND (“octreotide”[MeSH] OR “octreotide acetate” OR somatostatin analog) AND (randomized controlled trial [pt] OR controlled clinical trial [pt] OR randomized [tiab] OR placebo [tiab] OR clinical trials as topic [mesh: noexp] OR randomly [tiab] OR trial [ti])

Embase search from 1980 to November 2016
1 (pancreaticoduodenectomy* or PD* or pylorus-preserving pancreaticoduodenectomy* or PPPD* or peritoneoscop*).af.
2 exp pancreatic resection/

3 1 or 2
4 (pancreatectomy* or pancreectomy*).af.
5 exp pancreatectomy/
6 4 or 5
7 3 and 6
8 (octreotide or octreotide acetate or somatostatin analog).af.
9 exp octreotide/
10 8 or 9
11 (random* OR factorial* OR crossover* OR placebo*).af.
12 exp crossover-procedure/ or exp double-blind procedure/ or
exp randomised controlled trial/ or single-blind procedure/
13 11 or 12
14 7 and 8 and 13

SinoMed is a China biomedical literature service system, thus the search strategy is in Chinese: "奥曲肽"[全字段] OR "奥曲肽醋酸盐"[全字段] OR "奥曲肽"[主题词]) AND "胰腺切除"[全字段] AND "术后并发症"[全字段] AND "随机对照研究"[全字段]

Cochrane Library databases search from 1980 to November 2016

1 MeSH description of pancreatectomy explode all trees

2 (pancreaticoduodenectomy* PD* OR pylorus-preserving pancreaticoduodenectomy* OR PPPD*) AND pancreatectomy*

3 1 or 2

4 MeSH description of octreotide explode all trees

5 (octreotide acetate) OR (somatostatin analog) OR octreotide

6 4 or 5

7 3 AND 6
